# An Assessment of Human–AI Interaction Capability in the Generative AI Era: The Influence of Critical Thinking

**DOI:** 10.3390/jintelligence13060062

**Published:** 2025-05-26

**Authors:** Feiming Li, Xinyu Yan, Hongli Su, Rong Shen, Gang Mao

**Affiliations:** 1 Zhejiang Key Laboratory of Intelligent Education Technology and Application, Zhejiang Normal University, Jinhua 321004, China; 2 College of Education, Zhejiang Normal University, Jinhua 321004, China; y1253241738@163.com (X.Y.); 19557963637@163.com (H.S.); 3 School of Psychology, Zhejiang Normal University, Jinhua 321004, China; sr04282024@163.com

**Keywords:** human–AI interaction capability, information literacy, critical thinking

## Abstract

(1) Background: In the era of generative AI (GenAI), assessing AI literacy is essential for understanding how effectively non-expert users can interact with AI. However, existing assessment tools primarily focus on users’ understanding of AI principles or rely on self-reported scales, neglecting critical thinking and actual interaction capabilities. To address this gap, this study aims to design and validate evaluation indicators targeting the behavioral process of human–GenAI interactions and analyze the impact of critical thinking. (2) Methods: Grounded in information literacy and critical thinking frameworks, this study operationalized human–AI interaction capabilities into behavioral indicators and rubrics through observation, surveys, and pilot studies. Data were collected from 121 undergraduates completing two real-world tasks with GenAI, and their interaction processes were documented and evaluated. (3) Results: The indicators showed acceptable inter-rater and internal consistency reliability. Exploratory and Confirmatory Factor Analysis confirmed a three-dimensional structure. Further analysis showed that interaction capabilities varied across gender, academic background, AIGC use frequency, critical thinking disposition levels, and question chain logic. (4) Conclusions: The developed evaluation indicators are reliable and valid. Further analysis reveals that a high critical thinking disposition can offset the disadvantage of lower usage frequency. This highlights the significance of critical thinking in enhancing human–GenAI interaction capabilities.

## 1. Introduction

Since the release of GPT-3.5 by OpenAI, GenAI-related applications have been profoundly reshaping the paradigms of information generation, dissemination, and utilization. Compared to other AI technologies such as machine learning and natural language processing, GenAI can interact with humans in a more user-friendly manner, reduce the demands on human programming abilities, and effectively enhance the work efficiency of many computer-reliant tasks. Nonetheless, the passive acceptance of data outputs by human users, without a comprehensive grasp of the underlying architecture, operational principles, and information processing procedures of GenAI systems, entails significant risks of cognitive dependency (Ruan and Wang 2023). Some researchers have expressed concern that the use of GenAI may weaken various cognitive processes, including logical deduction and (Qiu and Li 2023) factual reasoning (Xing et al. 2024) and critical thinking (Azcárate 2024; Feng 2023), and may critically undermine users’ ability to think independently. Simultaneously, the mixture of true and false information (Jiao 2023; Mo et al. 2023) may impede users’ information processing capabilities. Moreover, GenAI systems may occasionally generate responses that exhibit biases and subjectivity. Therefore, the ability to represent problems, acquire and evaluate information, and obtain ideal answers during human–AI interaction, that is, human–AI interaction capability, becomes particularly important.

The study of human–AI interaction capability is an emerging research hotspot. Some studies assess learners’ abilities from an AI-centric perspective, using assessment frameworks that emphasize users’ understanding of the AI principles and operations (Liang et al. 2020). This approach not only overlooks human–GenAI interaction capability, but also underestimates the AI literacy of the broader non-expert user population. Some research emphasizes that human–AI interaction capacity is an important behavioral dimension of AI literacy (Miao and Holmes 2021). However, the measurement of this capacity remains based on self-reported scales (Yuan et al. 2024), which cannot accurately assess the true level of interaction ability based on evidence from actual human–AI interaction behaviors. Therefore, to more effectively assess the AI literacy of non-expert users, we need to design evaluation indicators and rubrics that focus on the process of human–GenAI interaction behavior.

Essentially, the interaction between humans and GenAI is akin to the interaction between humans and other types of media, as both aim to facilitate the efficient acquisition of accurate information. However, compared to traditional media, the anthropomorphism of GenAI—resembling an authoritative expert—may reduce human skepticism toward information, thereby making the involvement of critical thinking during interactions even more crucial. Therefore, beginning with traditional information literacy, this study analyzes the information processing procedures involved in human–GenAI interactions, develops a fundamental measurement framework for human–GenAI interaction competence, and establishes evaluation criteria based on the behavioral manifestations of critical thinking that permeate through the stages of information processing. On this basis, the study first develops evaluation tasks for assessing human–AI interaction capability and establishes an evaluation rubric. It then examines the validity of the assessment tools and, finally, investigates some influencing factors of human–GenAI interaction competence.

## 2. Theoretical Framework

### 2.1. Definition and Core Elements of Human–AI Interaction Capability

Typically, discussions of human–computer interaction (HCI) are technology-centric, with ‘computer’ broadly encompassing various technologies. In the Information Age, HCI primarily referred to individuals’ abilities to use computers for information acquisition and processing. In the Digital Age, HCI has evolved to focus on leveraging digital devices and technologies for these purposes. With the advent of intelligent technologies, there is now a requirement for humans to understand and effectively utilize smart information products and services. The interaction between humans and GenAI involves systematically posing questions to GenAI tools, learning from and reflecting on the feedback or generated text, formulating new questions, and evaluating and filtering the information produced by GenAI. The ultimate goal is to integrate GenAI responses to effectively solve real-world problems. The use of GenAI differs from that of a typical search engine, as it requires learners to provide coherent, clear, and precise prompts in order to obtain more valuable information (Huang et al. 2024a, 2024b). Furthermore, since there may still be errors in the content generated by GenAI, it is essential for learners to possess the ability to analyze and assess the quality of the information generated by GenAI. Therefore, this study defines Human–AI interaction capability (HAII-capability) as the ability to interact with generative AI products by applying prompt engineering (Bang et al. 2023; Lo 2023) techniques to retrieve, analyze, evaluate, and effectively integrate relevant information.

HAII-capability is intricately linked with concepts such as digital literacy and AI literacy, while also possessing distinct and unique attributes. In the Information Age, information literacy refers to the ability to use various information tools to acquire information resources and solve information-related problems (Zurkowski 1974; Zhang 1998). It encompasses elements such as information needs, acquiring information, analyzing information, using information, evaluating information, creating information, and ethics (ACRL Board 2016; American Library Association 2000; Bundy 2004; Zeng et al. 2006). In the era of intelligent technologies, AI literacy (Kong et al. 2021) refers to the ability to recognize and understand AI, utilize various AI technologies to accomplish tasks, analyze and select appropriate AI technologies and critically evaluate their outcomes, and acquire knowledge about the risks and ethical issues associated with AI (Wang et al. 2023a). Undoubtedly, intelligent interaction capability is an integral part of AI literacy, reflecting its behavioral aspects. This capability also inherits key competencies from information literacy, such as the ability to retrieve, analyze, evaluate, and integrate information. In addition, the abilities required for information literacy, such as the clarity, accuracy, relevance, and logicality of evaluating information, all depend on critical thinking. The *AI Competency Framework for Students*, published by the United Nations Educational, Scientific and Cultural Organization, states that critical thinking is crucial for students in the AI era, playing a pivotal role in AI literacy (Dan and Jing 2022). Given the significant unknowns and inaccuracies in information generated through human–GenAI interactions, empowerment through critical thinking is particularly vital.

### 2.2. The Nature and Assessment of Critical Thinking

To better assess HAII-capability, which includes critical thinking elements, we need to deepen our understanding of the nature of critical thinking and its assessment methods.

The concept of critical thinking has evolved from its philosophical roots in ancient Greek thought, particularly through the Socratic method of questioning (Wu 2014), to its modern emphasis on reflective and reasoned judgment. The American Philosophical Association gathered 46 experts and employed the Delphi method to discuss the nature and structure of critical thinking over several rounds. They reached a consensus that the dimensions of critical thinking skills include (a) interpretation, (b) analysis, (c) evaluation, (d) inference, (e) explanation, and (f) self-regulation (Facione 1990). Thus, critical thinking skills not only fully overlap with HAII-capability in the dimensions of analysis and evaluation, but also align with information integration through inference and explanation. Importantly, the self-regulation dimension of critical thinking offers key insights for measuring HAII-capability, emphasizing the need for humans to review processes and outcomes during GenAI interactions, continuously refine their prompts, and identify the most effective problem-solving approaches.

Early scholars emphasized logic and reasoning as key cognitive skills in critical thinking, which traditional tests often measured through objective questions (Bernard et al. 2008; Halpern 2007, 2013). Since the 21st century, however, critical thinking has been increasingly defined by its application in social, cultural, and real-world contexts. Reflecting this shift, recent assessments like the Collegiate Learning Assessment (CLA) and the iPAL critical thinking test have adopted performance-based tasks to evaluate critical thinking in real-world problem-solving scenarios (Aloisi and Callaghan 2018; Shavelson et al. 2018; Jiang et al. 2022). This approach also offers a new perspective for assessing HAII-capability. Specifically, it proposes that HAII should be evaluated through performance-based assessments in real-world contexts. The interaction between humans and GenAI (Generative AI) provides a natural environment for dynamically observing the critical thinking process, thus eliminating the need for extensive artificial setups and interventions by researchers. For example, there is no need to select large volumes of reading materials for participants or design specialized computer interaction platforms to observe the critical thinking process. Instead, it is only necessary to identify appropriate task scenarios that allow participants to freely interact with various pieces of information through GenAI.

Currently, most scholars agree that critical thinking includes critical thinking skills and a critical thinking disposition (Facione 1990; Ennis 1993; Dwyer et al. 2014). Facione et al. (1996) defined a critical thinking disposition as “the consistent internal motivation to engage problems and make decisions by using critical thinking”. In other words, a critical thinking disposition is an attitude, thinking habit, or intrinsic motivation that encourages individuals to use critical thinking skills to look at a problem, analyze it, and finally make a decision. Facione (1990) believed that skills and dispositions are two distinct aspects of critical thinking. The acquisition of critical thinking skills does not necessarily imply the concurrent development of a critical thinking disposition. However, a critical thinking disposition can predict the use of critical thinking skills. Since HAII (Human–Agent Interaction Intelligence) capability encompasses elements of critical thinking skills, this study uses critical thinking disposition as a criterion to validate the criterion-related validity of HAII-capability, while also examining the impact of critical thinking disposition and other factors on HAII-capability.

Facione (1990) developed the California Critical Thinking Disposition Inventory (CCTDI), with 75 items covering seven dimensions: Truth-seeking, Open-mindedness, Analyticity, Systematicity, Confidence, Inquisitiveness, and Maturity. Despite the widespread use of the CCTDI, it suffers from an excessive number of items, and unstable reliability and factor structure. Hou et al. (2022) developed a critical thinking questionnaire based on Byrnes and Dunbar (2014). It includes three dimensions: analytic skills, open-mindedness, and the tendency to apply critical thinking. Although the questionnaire claims to integrate skills and dispositions, its self-report format means it primarily measures dispositions. This study used it to measure critical thinking disposition due to its clear dimensions and cultural relevance.

Combining the perspectives of information literacy and critical thinking, HAII-capability is further defined as the ability to critically retrieve, analyze, evaluate, and integrate information during human interaction with GenAI while continuously scrutinizing the process and outcomes to regulate one’s behavior. However, existing assessment tools primarily focus on users’ understanding of AI principles or rely on self-reported scales, neglecting critical thinking and actual interaction capabilities. To address this gap, this study aims to design and validate evaluation indicators targeting the behavioral process of human–GenAI interactions and analyze the impact of critical thinking. Therefore, this study primarily addresses three questions:

**Question** **1:** *What is the framework of HAII-capability? How can an effective assessment method be designed?*

**Question** **2:** *Is the assessment of HAII-capability reliable and valid?*

**Question** **3:** *What factors influence HAII-capability, and is it related to critical thinking?*

## 3. The Rubric for the Iterative Generation of HAII-Capability

First, the definition of HAII-capability essentially establishes its framework, which primarily draws from the four stages of information processing: retrieving, analyzing, evaluating, and integrating information. However, when observing the interactive process, we found that the stages of acquiring and analyzing information are always intertwined in interactions with GenAI, as the act of retrieving information is always accompanied by the analysis of the preceding information. Therefore, we ultimately established a three-dimensional framework: information retrieving and analysis, information evaluation, and information integration. Further, each dimension is described in Table 1, including essential steps in the process of humans leveraging AI to acquire information and solve problems.

The establishment of the criteria underwent a multi-stage iterative process: (1) Based on the theoretical definitions of the above dimensions, a brainstorming session within the research team was conducted, drawing on individual experiences: What specific behavioral manifestations of critical thinking and interaction skills can be observed in each dimension (interaction stage)? Where do the differences in these behaviors lie? (2) We collected interaction traces of university students as they used GenAI to complete daily activities or learning tasks. We used these initially established criteria and rubrics to assess their adaptability, sensitivity, and relevance to the measurement objectives while identifying key behaviors not covered by the criteria and supplementing them with additional criteria targeting those behaviors. (3) We integrated feedback from the research team on the indicators to develop a more mature set of indicators and rubrics, and conducted testing based on the tasks set for the assessment. (4) We analyzed the discrimination of each criterion in the scoring results, interviewed the participants and raters, and gathered their suggestions on the rationality of the criteria and rubric design.

After four stages of iteration, indicators that were not central to HAII-capability were removed, such as those related to “Generating innovative questions”. Next, indicators with low discriminatory power were discarded, for instance, “clearly stating one’s viewpoint” and “summarizing and synthesizing GenAI’s responses during the Q&A process”. The former was achievable by nearly all participants, while the latter was rarely performed. The final set of indicators is presented in Table 2, and the rubric is provided in Appendix A, Table A1.

## 4. Task Design and Research Method

### 4.1. Task Design

After establishing the criteria, the central challenge in task design is to determine which tasks can most effectively prompt participants to exhibit the behaviors outlined in the criteria during their interactions with GenAI. Additionally, the tasks should be structured to highlight significant behavioral differences among participants with varying interaction abilities. The interaction between humans and GenAI is fundamentally an activity of knowledge construction. Wang and Zhong (2008) proposed that task design supporting adaptive knowledge construction activities should include characteristics such as authenticity, ill-structuredness, complexity, challenge, and experientiality. These characteristics generally apply to our task design, with the exception of experientiality. Assessment tasks, unlike typical activity tasks, are designed to minimize the influence of participants’ prior experiences on their performance. In alignment with the objectives of this study, we have summarized three main principles: ill-structuredness, authenticity, and limited experientiality.

(1). Ill-structuredness. Ill-structuredness serves as a core design principle because an ill-structured problem inherently embodies both complexity and challenge. Ill-structured problems are those on which opposing or contradictory evidence and opinions exist, for which there is not a single, correct solution that can be determined by employing a specific decision-making process (Kitchner 1983). Jonassen (2000) distinguished between well-structured and ill-structured problems, proposing 11 types of problems with varying characteristics. These problems range from well structured to ill structured as follows: logical problems, algorithmic problems, story problems, rule-using problems, decision making problems, trouble-shooting problems, diagnosis-solution problems, strategic performance problems, case analysis problem, design problems, and dilemmas. Therefore, in the task design of this study, tasks similar to dilemmas were chosen as much as possible, to ensure that participants could directly obtain the final answer from GenAI, thus providing space for sufficient interaction with GenAI (Ku 2009).
(2). Authenticity. Authentic problems hold greater intrinsic value and significance for humans, thereby more readily stimulating interest in their resolution. To ensure task authenticity, this study selected one problem from the social domain and one from the scientific domain, both drawn from existing and relevant problems in society and life.
(3). Limited experientiality. Whenever a problem is authentic, it is challenging to prevent participants from drawing on their prior experiences and existing knowledge. Therefore, it is impossible to completely exclude the influence of participants’ varying experiences on their interactive performance. However, when selecting tasks, a balance must be struck. On one hand, participants should have some prior knowledge of the problem to serve as a starting point for exploration; on the other hand, most participants should not have extensive experience with the problem, ensuring there is room for further exploration and allowing us to fully assess their ability to interact with GenAI through their interactive behaviors.
  Based on the above criteria, this study selected one scientific problem scenario and one social problem scenario, and developed specific task requirements, as detailed in Table 3.

### 4.2. Research Method

#### 4.2.1. Participants and Procedure

This study recruited 121 undergraduate and graduate students from various disciplines at two universities. For this assessment, participants were provided with two scenario-based tasks via a Word document. Each task included a problem scenario and specific task requirements. After reading and understanding these requirements, all participants were systematically instructed to utilize Tongyi Qianwen 2.5 (Qwen-2.5), a Chinese-optimized LLM, to address Mandarin-specific linguistic challenges including lexical ambiguity, syntactic complexity, and cultural-context adaptation. They were then required to submit the complete interaction process along with their final problem-solving responses. Additionally, participants completed a critical thinking questionnaire via a survey website. Students were required to independently complete all tasks within a strict 45 min time limit. Participants provided their written consent, and this study was approved by the ethics committee of Zhejiang Normal University under the Declaration of Helsinki.

#### 4.2.2. Materials

In addition to the assessment tasks in Appendix A Table A1 and the evaluation rubric in Appendix A Table A1, which serve as the primary tools for evaluating HAII-capability, this study also included a questionnaire that collected participants’ background information (such as gender, major, grade level, and frequency of GenAI use) and a critical thinking questionnaire. Based on the hypothesis that HAII-capability is correlated with levels of critical thinking, critical thinking was selected as the criterion for assessing HAII-capability. To minimize the impact of cultural differences, this study employed a culturally adapted critical thinking scale developed by Hou et al. (2022). This scale includes 17 items across three dimensions: Openness, Analytical Skills, and Application. The overall reliability of the scale is 0.89, with subscale reliabilities of 0.77 for Openness, 0.91 for Analytical Skills, and 0.77 for Application.

## 5. Results

### 5.1. Reliability

To minimize errors due to subjectivity in scoring, this study recruited six raters and provided them with training, including a detailed introduction to the purpose, framework, scoring criteria, and rubrics of the assessment tool, with a focus on discussing ambiguous or vague scoring criteria and rubrics. Only after all raters reached a unified understanding of the scoring criteria did the researchers assign 10 identical sets of subject data to the six raters for back-to-back scoring. Based on the Intraclass Correlation Coefficient (ICC) shown in Table 4, the inter-rater reliability of the six raters was 0.756. After thorough discussions on the criteria with significant discrepancies among the 10 subject cases, in order to reduce scoring time, the six raters were evenly divided into three groups, with each group assigned 37 different subject data for back-to-back scoring. Thereafter, the reliability between each pair of raters within each group was calculated separately. Based on the Intraclass Correlation Coefficient (ICC), the results indicated that the three groups of raters had desirable inter-rater reliability, with scores of 0.916, 0.851, and 0.765, respectively. This suggests that all raters achieved a relatively consistent understanding when applying the rubric for scoring, ensuring the comparability of the subjects’ scores across various indicators. At the same time, the internal consistency reliability of all criteria and the three sub-dimensions was obtained by calculating Cronbach’s α, as shown in Table 5, with all reliabilities above 0.75. Additionally, since the assessment tool consisted of two tasks, the correlation in performance between the science and social themes, serving as an indicator of alternative-form reliability, was 0.52, indicating a moderate correlation between the two tasks. This suggests that the consistency between the two tasks was not high.

### 5.2. Validity

The validity evidence in this study was primarily derived from construct validity and criterion validity. The analysis of construct validity was conducted using Exploratory Factor Analysis (EFA) and Confirmatory Factor Analysis (CFA). First, based on the scree plot from the EFA, it was determined that there are three factors. Indicators with loadings below 0.4, such as U1 (the number of effective questions raised), were removed. The final factor structure, a three-factor structure, is shown in Table 6.

**Figure 1:**
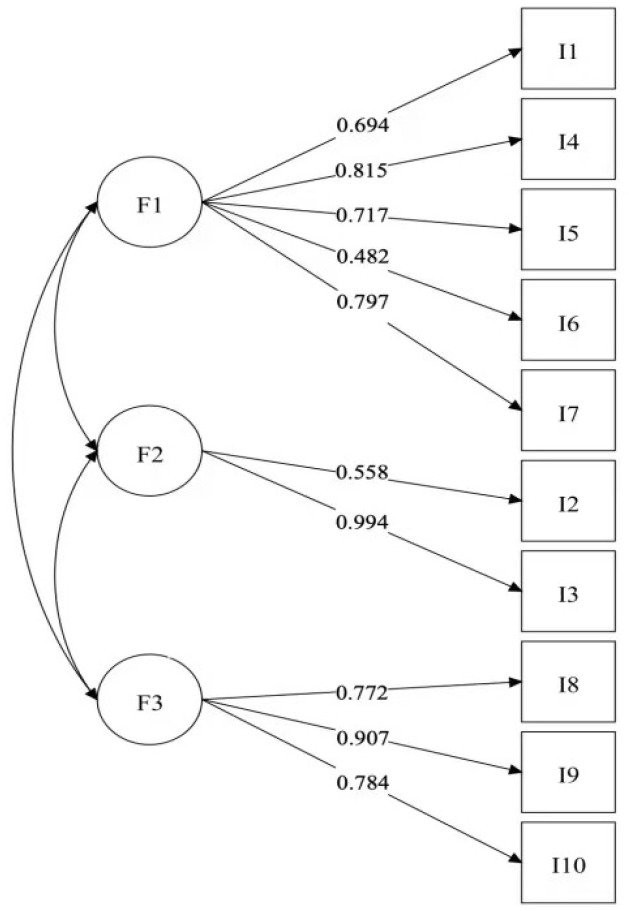
CFA model of HAII-capability.

Construct validity was assessed through Confirmatory Factor Analysis (CFA), where a comparison was made between a single-factor model and the proposed three-factor model. The model fit indices for both are presented in Table 7. For the three-factor model, the χ^2^/df was 1.34, the Comparative Fit Index (CFI) was 0.975, and the Tucker–Lewis Index (TLI) was 0.965. Additionally, the Root-Mean-Square Error of Approximation (RMSEA) was 0.053, and the Standardized Root-Mean-Square Residual (SRMR) was 0.068. All fit indices met acceptable thresholds, and the fit of the three-factor model was significantly superior to that of the single-factor model. Figure 1 illustrates the CFA model’s structural representation, with factor loadings for each indicator on its respective dimension exceeding 0.4, most of which were above 0.6, indicating strong alignment between indicators and their dimensions. Moreover, the correlation matrix between the three dimensions revealed low correlations between any two dimensions, suggesting that each dimension represents a relatively independent aspect of HAII-capability. Collectively, these findings provide robust support for the validity of the proposed three-factor model in this study.

The analysis of criterion-related validity selected critical thinking disposition as the criterion for concurrent validity. The correlations between critical thinking disposition and the total score of HAII-capability, as well as its subdimensions, are shown in Table 8. A significant correlation was found between critical thinking disposition and the overall HAII-capability level, as well as the information retrieval and analysis dimension. This suggests that university students with a higher tendency for critical thinking are more likely to critically retrieve and analyze information through effective questioning strategies.

### 5.3. Overview and Influencing Factors of Students’ HAII-Capability

**Figure 2:**
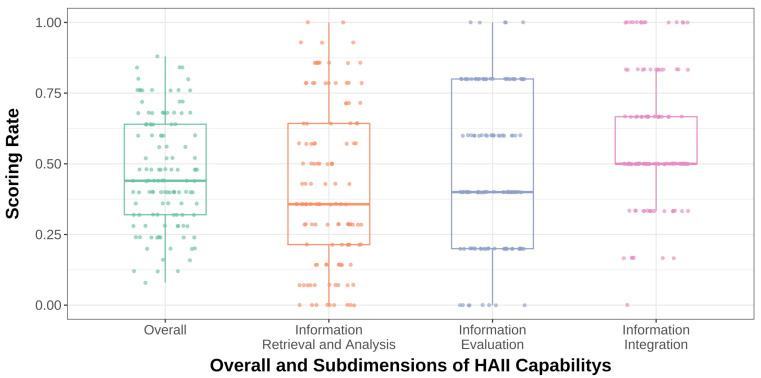
Box plot of students’ score rates in the three dimensions of HAII-capability.

Figure 2 shows box plots of the participants’ performance across the three dimensions. To ensure comparability, all data were normalized into a score rate (ranging from 0 to 1), calculated by dividing the dimension score by the total possible score for that dimension. The figure reveals that students exhibit the lowest average performance and the greatest variability in Dimension 1, “Information Retrieval and Analysis”. In contrast, Dimension 3, “Information Integration”, has a relatively higher average performance and the smallest variability. Dimension 2, “Information Evaluation”, falls between the two in terms of both average performance and variability.

To further explore the factors influencing students’ HAII-capability, this study compares differences in HAII-capability and its three dimensions based on gender, discipline, usage frequency (see Table 9), and questioning logic structures (see Table 10). Regarding gender, a significant difference is found only in Dimension 2, “Information Evaluation”, where male students are more inclined to question the reliability and plausibility of the information provided by GenAI, demonstrating a better ability to evaluate the quality of GenAI responses. The comparison between science and humanities students shows that, with the exception of Dimension 3, “Information Integration”, science students perform significantly better than humanities students in other dimensions and in the overall HAII-capability assessment. Regarding usage frequency, since only six participants reported “never using” the tool, the “never used” group was combined with the “less than or equal to once a week” group. The results show that, with the exception of Dimension 3, “Information Integration”, the higher the frequency of GenAI usage, the stronger the HAII-capability. This suggests that experience of interacting with GenAI enhances our understanding of its performance, thereby improving HAII-capability.

Understanding how to effectively communicate with GenAI to obtain accurate information in the most efficient manner is one of the most crucial dimensions of HAII-capability. As such, the logical structure of the questions posed serves as a key influencing factor. Based on the analysis of the sequence and hierarchical relationships of questions posed during participants’ interactions with GenAI, the raters categorized the logical structure of participants’ questions into three types of questioning logic: a Fully Parallel mode, a fully progressive mode, and an Initially Parallel then Progressive mode. Since the three types of questioning logic may vary across different tasks for each participant, we conducted the analysis separately for each task. The results revealed significant differences in HAII-capability and Dimension 1, “Information Acquisition and Analysis”, across the three types of questioning logic. The ‘Initially Parallel Then Progressive’ questioning logic significantly outperformed the other two types. Additionally, the ‘Fully Progressive’ questioning logic was significantly superior to the ‘Fully Parallel’ type in scientific issues.

**Figure 3:**
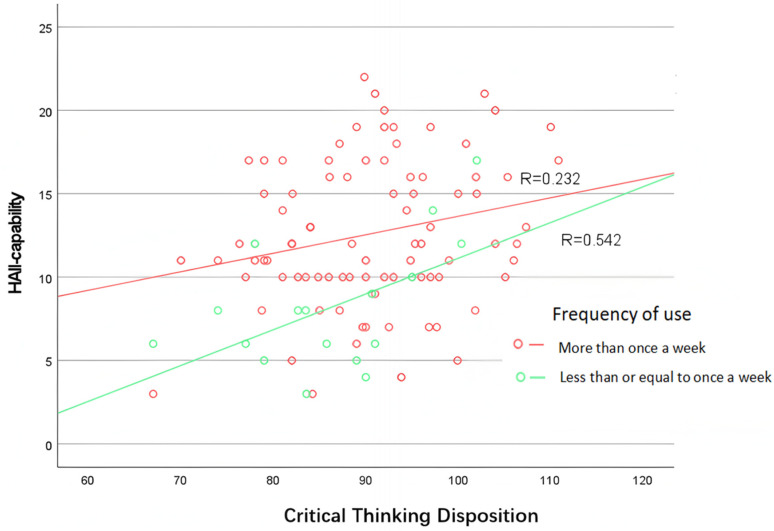
Scatter plot of HAII-capability and critical thinking disposition across different GenAI usage frequencies.

Critical thinking disposition, as the most central influencing factor of HAII-capability, has already been shown to have a significant correlation with HAII-capability. Furthermore, the comparative analysis above indicates that the frequency of GenAI usage also impacts HAII-capability. A related practical issue is whether the frequency of GenAI usage can moderate the influence of critical thinking disposition on HAII-capability. To address this question, a scatter plot (Figure 3) was created with critical thinking disposition on the X-axis and HAII-capability on the Y-axis. The plot reveals that, on the one hand, at the same level of critical thinking disposition, the HAII-capability of the “more than once a week” group is consistently higher than that of the “Once a week or less” group. However, when the critical thinking disposition score reaches around 120, the HAII-capability levels of both groups converge. This suggests that students with sufficiently high critical thinking abilities are capable of exhibiting strong interaction with GenAI, even with minimal use of GenAI. From another perspective, the slope of the “more than once a week” group is lower than that of the “Once a week or less” group. This suggests that critical thinking disposition has a greater impact on HAII-capability for students with lower usage frequency. An increase in usage frequency can mitigate the impact of insufficient critical thinking disposition on HAII-capability.

Furthermore, the scatter plot in Figure 3 illustrates that critical thinking disposition and usage frequency jointly influence HAII-capability. At the starting point, individuals with the same level of critical thinking disposition tend to have higher HAII-capability if they use GenAI more frequently. As critical thinking disposition levels increase and reach around 120, individuals with both high and low usage frequencies converge to similar levels of HAII-capability. This suggests that a sufficiently high critical thinking disposition can compensate for the disadvantage associated with lower usage frequency, indicating that both factors play a combined role in influencing HAII-capability.

## 6. Discussion

The first research question of this study pertains to how an effective assessment method can be designed. To address this question, the study first established a framework for HAII-capability, developed relevant behavioral criteria and rubrics, and designed tasks to stimulate interactive behaviors. Starting from the essence of human–computer interaction, this study first analyzes HAII-capability, which, as a behavioral aspect of AI literacy, shares a deep-rooted connection with information literacy. This forms the foundation for its basic framework, which includes the retrieval, analysis, evaluation, and integration of information. However, on the other hand, the GenAI environment upon which HAII-capability relies introduces greater potential for confusion. For example, intelligent tools tend to provide answers that appear confident even when they lack the necessary information (Heersmink et al. 2024). Therefore, critical thinking must be integrated throughout the entire process of intelligent interaction, from information processing to application. The assessment of HAII-capability requires incorporating critical thinking skills into its criteria design. In this regard, this study fully considers the rational analysis, questioning, and evaluation of information, as well as the review of the entire interaction process and its outcomes, continuously adjusting prompt behaviors and strategies in the criteria design.

To better evaluate HAII-capability, this study compares and analyzes existing methods of assessing critical thinking. It finds that observing and evaluating the behavior of human–GenAI interactions in real-world problem-solving contexts may yield more authentic and reliable assessment results than traditional self-report scales. How can we obtain behavioral evidence reflecting HAII-capability from the extensive human–GenAI interactions across different task contexts? On the one hand, we need to identify cross-task invariant behavioral criteria and establish corresponding rubrics. On the other hand, we must select representative assessment tasks to ensure that the impact of different task types on participants’ performance is minimized. The establishment of behavioral criteria and corresponding rubrics is based on the research team’s observation and synthesis of extensive real-world GenAI interaction behaviors, feedback from both participants and raters regarding the tests, and the analysis of pre-test data. The iterative optimization across multiple stages helped us identify the indicators that are truly relevant to HAII-capability and have strong discriminatory power. This iterative approach has been proven to be a very effective way of selecting behavioral evidence among massive process data.

The second research question pertains to whether the assessment of HAII-capability is reliable and valid. How well behavior can be assessed sometimes depends on the quality control of administration, such as the rating procedure among multiple raters. The training helped raters understand the rubrics, and the discussion on inconsistent ratings helped improve consensus. As long as the inter-rater reliability was ensured, the reliability of total and sub-dimensions of HAII-capability reached above 0.7, which is ideal for behavior measures. EFA and CFA both confirm that a three-factor structure is the best-fitting model, which somewhat deviates from our initial assumption of a four-factor structure being the best. Its main difference compared to the theoretical four-factor model is that the dimensions of information retrieval and analysis have been combined into a single dimension. This may be related to the iterative process of information retrieval–analysis–retrieval in human–GenAI interactions, where the process of retrieving new information implicitly involves analyzing the previous information before acquiring the next. The convergence of information retrieval and information analysis reflects the characteristics of interacting with GenAI.

The third research question pertains to what factors influence HAII-capability and whether it is related to critical thinking disposition. This article analyzes this from the perspective of both internal cognitive and external factors. Internal cognition includes two factors: critical thinking disposition and types of questioning logic. Existing research shows that critical thinking is playing an increasingly important role in information literacy. The skills required for assessing the clarity, accuracy, and logical strength of information, which are integral to information literacy, all depend on critical thinking (Paul and Elder 2008). The HAII-capability proposed in this study may also be influenced by critical thinking disposition. At the same time, whether generative AI products, represented by ChatGPT, will impact the enhancement of students’ critical thinking skills has sparked widespread attention and debate within the academic community. Some researchers argue that ChatGPT can promote students’ critical thinking, reading, and writing skills (Wang et al. 2023b; Zhou et al. 2024), while others suggest that ChatGPT may diminish students’ ability to critically analyze and filter information, hindering the development of their critical thinking skills (Else 2023). These contrasting perspectives indicate that there may be a complex relationship between GenAI and critical thinking. Therefore, this study analyzes critical thinking disposition as a factor influencing HAII-capability. The results indicate that individuals with a stronger critical thinking disposition tend to score higher in HAII-capability, and this may even make up for their lack of competence in GenAI usage. This suggests that critical thinking disposition plays a crucial role in interactions with GenAI. Those with higher levels of critical thinking disposition are more likely to approach GenAI-generated responses with a scientific and rational attitude, forming their own unique insights and systematically and logically engaging with GenAI through prompts. This result, to some extent, indicates that the tasks designed in this study stimulated students’ critical thinking; otherwise, all students would have arrived at the same and “only correct” answer. Therefore, an important implication of this study is that when teachers cannot prevent students from using GenAI but still wish to develop students’ higher-order thinking, they need to consider task characteristics that can fully stimulate students’ critical thinking, as this study followed the principles of ill-structured, authentic, and limited-experience tasks. As suggested by some research (van den Berg and du Plessis 2023), ChatGPT-generated resources can serve as a starting point for students’ critical analyses. Thus, the tasks assigned by teachers need to go beyond simply acquiring resources and answers and should instead include more open-ended and ill-structured problems to encourage students’ critical thinking (Bower et al. 2024).

Regarding the logic structure of questioning (types of questioning logic), this study first reviews the findings from existing research. Ramos (2009), drawing from a hierarchical framework of research questions, suggests that well-formulated research questions should exhibit either a sequential or hierarchical distribution. Drawing on previous relevant studies, this research proposes three questioning modes: Fully Parallel, Fully Progressive, and Initially Parallel Then Progressive. The Fully Parallel structure tends to be more dispersed and random in its questioning. Although it covers a broad range of content, the logical connections between the questions are relatively weak. In contrast, the Fully Progressive structure emphasizes the logical connections between questions or between questions and answers, progressively delving deeper into the topics, resulting in more in-depth coverage. The third type, Initially Parallel Then Progressive, not only focuses on breadth, but also emphasizes the depth of questioning in the details, providing more comprehensive consideration of the issues. Therefore, it can be observed that individuals using the Initially Parallel Then Progressive mode tend to score higher on HAII-capability, followed by those using the Fully Progressive mode, with the lowest scores attributed to those using the Fully Parallel mode. To a certain extent, the logic of the structure reflects one’s critical thinking disposition when monitoring the process of interacting with GenAI. Thus, the relationship between the logic structure of questioning and HAII-capability provides additional evidence supporting the influence of critical thinking disposition on HAII-capability.

This study focuses on constructing an assessment framework for Human–AI Interaction (HAII) capability and conducting a comprehensive evaluation of university students’ HAII competencies. While the research outcomes largely align with our expectations, several limitations warrant acknowledgment.

First, regarding the methodological constraints, our study encounters two primary challenges: (1) the limited sample size and representativeness that fails to comprehensively encompass students across all academic years, and (2) the restricted selection of task themes (limited to the science and society domains), which may introduce bias and not fully capture HAII-capability in real-world applications. To address these limitations, subsequent research should expand both the sample diversity (including participants from various disciplines, cultural backgrounds, and educational levels) and the range of task themes while systematically collecting data on students’ actual usage of generative AI in daily contexts.

Second, concerning the measurement limitations, we identify two critical issues: (1) The current three-dimensional assessment framework evaluates human–AI interaction primarily through an information literacy perspective, revealing two cognitive measurement constraints. The information acquisition dimension’s dependence on Bloom’s taxonomy effectively measures comprehension and application but lacks robust metrics for higher-order cognitive processes like adaptive decision-making. Furthermore, the systematic assessment of metacognitive strategies remains unaddressed. (2) While critical thinking disposition (CTD) serves as a foundational element of critical thinking constructs and demonstrates empirically validated correlations with HAII competencies—specifically regarding critical information vetting and evidence-based reasoning capacities—the prevailing dependence on self-reported CTD instruments introduces measurable validity threats. These psychometric limitations stem not only from inherent social desirability response bias but also, more fundamentally, from their incapacity to fully capture students’ authentic critical thinking application in practical tasks. Future research should incorporate validated measures of both executive functions (e.g., problem-solving heuristics) and metacognitive awareness to more comprehensively assess the complexity of human–AI cognitive collaboration. Additionally, integrating behavioral data (e.g., task performance metrics) and other objective measurement methodologies (e.g., simulated scenarios) would establish more accurate and holistic measures for assessing students’ critical thinking skills, thereby providing empirical criteria for validating HAII capabilities through criterion-related validity evidence.

Finally, the absence of a direct comparative analysis between human–AI collaboration outcomes and expert decisions limits our capacity to assess the efficacy of AI-assisted problem-solving against established benchmarks. Potential extensions of this research could incorporate expert decisions as reference models to establish rigorous benchmarks for assessing human–AI collaboration while simultaneously exploring hybrid models that synergize human expertise with AI capabilities to deepen our understanding of their complementary roles in complex problem-solving scenarios.

## Figures and Tables

**Table 1:** A description of the HAII-capability framework.

	HAII-Capability
Information Retrieval	Be able to analyze the initial information, query AIGC for information, clearly comprehend the meaning conveyed by the information, and promptly follow up when something is not understood. During the interaction, continuously identify the gap between the required information and the information provided by AIGC, and adaptively query AIGC to obtain further information.
Information Analysis	
Information Evaluation	Be able to critically evaluate the relevance, credibility, and logical strength of the information and assess whether the information has omissions or biases
Information Integration	Be able to extract, filter, and synthesize the relevant main points from the information provided by AIGC according to the requirements of the task, and summarize (or organize) the information or draw reasonable conclusions based on the existing claims and evidence provided by AIGC, combined with one’s own ideas, from multiple aspects and with complete logic.

**Table 2:** Dimensions and criteria of HAII-capability.

Dimensions	Criteria
F1: Information Retrieval and analysis	U1: Number of valid prompts
	U2: Cognitive levels of questions
	U3: Multi-perspective prompts
	U4: Follow-up inquiries on GenAI responses
	U5: Continuously refining prompts based on GenAI responses
	U6: Prompt chain exhibits structure and logical relations
F2: Information evaluation	U7: Frequency and quality of justified queries
	U8: Critically evaluating and substantiating relevance, credibility, and logical strength in GenAI responses
F3: Information integration	U9: Screening and refining evidence from information
	U10: Justifying from multiple perspectives
	U11: Logical consistency of argumentation

**Table 3:** Tasks and prompts for assessing HAII-capability.

Problem Domain	Problem Scenario	Prompts
Social issues	During this year’s Two Sessions, the representatives of the National People’s Congress made many suggestions, such as ‘suggesting the establishment of an electronic medical record platform’ to unify patient cases from across the country into a comprehensive platform. The public has also been actively discussing this. Please communicate with ChatGPT to gather as much information as possible and complete the following tasks.	1. Based on its response and your own contemplation, decide for yourself whether to support this issue and provide reasons
		2. Evaluate the relevance, credibility, and logicality of ChatGPT’s responses, and please provide examples to illustrate your points.
Scientific issues	If there is a myopic patient, the currently known treatment methods include Femtosecond Laser, Femtosecond-assisted LASIK, Excimer Laser, and Phakic Intraocular Lens Implantation. Please communicate with ChatGPT to gather as much information as possible and complete the following tasks	1. Choose a treatment method that minimizes bodily harm, has the fewest subsequent side effects, and has a low probability of recurrence, or do not treat at all, and explain the reasons for your choice.
		2. Assess the relevance, credibility, and logicality of ChatGPT’s responses, and illustrate with examples

**Table 4:** Inter-rater reliability.

Six Evaluators	Evaluators 1 and 2	Evaluators 3 and 4	Evaluators 5 and 6
0.756	0.916	0.851	0.765

**Table 5:** Internal consistency reliability of scoring rubric.

Total	Information Retrieval and Analysis	Information Evaluation	Information Integration
0.75	0.816	0.716	0.713

**Table 6:** Standardized factor loadings of indicators for each dimension.

F1: Information retrieval and analysis	U2	0.734		
	U3	0.776		
	U4	0.733		
	U5	0.503		
	U6	0.778		
F2: Information evaluation	U7		0.768	
	U8		0.611	
F3: Information integration	U9			0.697
	U10			0.790
	U11			0.854

**Table 7:** Model fit indices for one-factor and three-factor CFA models.

Models	χ^2^/DF	CFI	TLI	RMSEA	SRMR
One-factor	6.37	0.57	0.44	0.21	0.18
Three-factor	1.34	0.975	0.965	0.053	0.068

Note. CFI = Comparative Fit index; TLI = Tucker–Lewis index; RMSEA = Root-Mean-Square Error of Approximation; SRMR = Standardized Root-Mean-Square Residual; DF = degree of freedom.

**Table 8:** Validating HAII capability: critical thinking as criterion via global and subscale correlations.

	Information Retrieval and Analysis	Information Evaluation	Information Integration	Total
Critical thinking disposition	0.355 **	0.14	0.043	0.317 **

Note. ** *p* < 0.01.

**Table 9:** Analysis of factors influencing HAII-capability.

	Information Retrieval and Analysis		Information Evaluation		Information Integration		Total	
	*M ± SD*	*t*	*M ± SD*	*t*	*M ± SD*	*t*	*M ± SD*	*t*
Gender								
Male	6.0 ± 3.9	0.1	3.0 ± 1.2	4.2 **	3.6 ± 1.4	0.8	12.6 ± 5.0	1.4 *
Female	5.9 ± 3.9		2.0 ± 1.2		3.3 ± 1.3		11.3 ± 4.6	
Discipline								
Science	6.6 ± 3.9	2.6 **	2.6 ± 1.4	2.2 *	3.4 ± 1.5	0.1	12.8 ± 5.0	2.8 **
Humanities	4.8 ± 3.6		2.0 ± 1.0		3.4 ± 1.1		10.3 ± 3.9	
Usage Frequency								
>Once a week	6.5 ± 3.7	4.1 **	2.5 ± 1.3	2.6 *	3.4 ± 1.3	−0.2	12.5 ± 4.6	4.1 **
<=Once a week	2.8 ± 3.2		1.7 ± 0.9		3.5 ± 1.2		8.0 ± 3.5	

Note: * *p* < .05 ** *p* < .01.

**Table 10:** Differences in HAII-capability and sub-dimensions based on questioning logic structures.

	F1		F2		F3		Total	
	*M ± SD*	*F*	*M ± SD*	*F*	*M ± SD*	*F*	*M ± SD*	*F*
Task 1: (Social Issues)								
Fully Parallel	5.1 ± 4.4	5.5 **	4.6 ± 2.6	0.5	2.6 ± 1.6	3.8 *	12.3 ± 5.2	6.3 **
Fully Progressive	5.7 ± 3.7		4.7 ± 3.2		3.1 ± 1.7		13.5 ± 6.4	
Initially Parallel then Progressive	8.0 ± 3.9		5.2 ± 3.3		3.6 ± 1.7		16.8 ± 6.8	
Task 2: (Scientific Issues)								
Fully Parallel	7.1 ± 4.5	8.8 **	4.7 ± 2.5	1.8	2.5 ± 1.6	0.4	14.4 ± 6.2	7.7 **
Fully Progressive	9.5 ± 3.2		4.6 ± 3.1		2.9 ± 1.6		17.0 ± 4.2	
Initially Parallel then Progressive	10.6 ± 3.5		5.7 ± 2.8		2.5 ± 1.7		19.0 ± 5.0	

Note: * *p* < .05 ** *p* < .01.

## Data Availability

The data generated and analyzed in this study are available from the corresponding author on reasonable request.

## Appendix A

**Table A1:** Rubrics for assessing HAII-capability.

Dimension	Scoring Rubrics	0	1	2	3
F1: Information Retrieval and Analysis	U1: Number of valid prompts	Up to three prompts	Between 3 and 5 prompts	Between 5 and 10 prompts	At least 10 prompts
	U2: Cognitive levels of questions	All prompts focus on low-order thinking such as knowledge, comprehension	1 to 2 high-order prompts, such as application, analysis, synthesis, and evaluation	More than 3 high-order prompts	Most of prompts are higher-order cognitive levels.
	U3: Multi-perspective prompts	—	Prompts from 1 to 2 perspectives	Prompts from 3 to 5 perspectives	Prompts from more than 5 perspectives
	U4: Follow-up inquiries on GenAI responses	No follow-up inquiries on GenAI responses	Follow-up inquiries into basic factual knowledge	Follow-up inquiries stemming from deliberate analysis and reasoning	—
	U5: Continuously refining prompts based on GenAI responses	The prompt does not undergo any adjustments	There are 1 to 2 adjustments made	There are more than 3 adjustments made	—
	U6: The prompt chain exhibits structure and logical relations	There is no logical relationship between the prompts	Prompts exhibit 1 to 3 instances of logical relationships	Prompts exhibit 3 to 5 instances of logical relationships	Prompts exhibit more than 5 instances of logical relationships
F2: Information Evaluation	U7: Frequency and quality of justified queries	Obvious errors in GenAI responses go unqueried	No room for queries in GenAI responses/selective query	Querying and analysis take place	—
	U8: Critically evaluating and substantiating relevance, credibility, and logical strength in GenAI responses	No evaluation of relevance, credibility, and logical strength	Evaluation of three aspects without justification	Evaluation of three aspects with partial justification	Evaluation of three aspects with full justification and examples
F3: Information Integration	U9: Screening and refining evidence from information	Conclusions are drawn without any supporting evidence	The GenAI response is copied without selecting fully relevant evidence to support the conclusion	Evidence is carefully selected from the GenAI response to ensure the necessity and relevance of the arguments	—
	U10: Justifying from multiple perspectives	Only one aspect is considered	Analyzing multiple aspects, but with limited depth	Multiple aspects are thoroughly and comprehensively analyzed	—
	U11: Logical consistency of argumentation	Direct copying of GenAI response without independent reasoning	Superficial processing with logical gaps	Refined processing with a complete logical chain	—
